# Models of care for people with dementia approaching end of life: A rapid review

**DOI:** 10.1177/02692163231171181

**Published:** 2023-05-07

**Authors:** Suzanne Lewis, Zoi Triandafilidis, Cassie Curryer, Sarah Yeun-Sim Jeong, Nicholas Goodwin, Sally Carr, Daneill Davis

**Affiliations:** 1 Central Coast Local Health District, Gosford, NSW, Australia; 2 Central Coast Research Institute (CCRI) for Integrated Care, University of Newcastle, Callaghan, NSW, Australia; 3 School of Nursing, University of Sydney, Sydney, NSW, Australia

**Keywords:** Dementia, palliative care, integrated care, patient-centred care, review, model of care, end-of-life care

## Abstract

**Background:**

People with dementia have different care and support needs at their end of life compared to people with other life-limiting illnesses, and general palliative care models may not meet the needs of people with dementia and their carers and families. Some dementia-specific end-of-life care models have been implemented, and a summary of existing models was undertaken to inform development of a local model.

**Aim:**

To identify best-practice models of care for people in the advanced and end stages of dementia, and their families and carers.

**Design:**

A rapid review with narrative summary of peer-reviewed articles and grey literature was conducted.

**Data sources:**

Ten databases were searched for articles published between January 2000 and April 2022. Inclusion criteria were: all care settings; AND the model focuses on people with end-stage or advanced dementia; AND contained multiple components.

**Results:**

Nineteen articles or reports, describing twelve dementia-specific models of end-of-life care in a range of care settings were identified for inclusion in the review. There is strong evidence that the principles of best practice palliative care for people with advanced dementia are well known, but limited examples of translation of this knowledge into integrated models of care. The key issues that emerged from the findings were: referral and admission to care, integration of care, sustainability and evaluation.

**Conclusions:**

Findings can be used to inform development of improved end-of-life care pathways for people with dementia, but well-designed research studies are needed to evaluate the effectiveness of integrated models of care for this vulnerable population.

**What is already known about the topic?**

- A palliative care approach is beneficial for people with advanced or end-stage dementia and the best practice components to guide such approaches are well known.
- Few models of end-of-life care to meet the needs of people with dementia have been implemented, and generic models may not be appropriate or responsive to their needs.
- Dementia-specific models of end-of-life care are required to meet the needs of growing numbers of people with dementia, and their families and carers.

**What this paper adds?**

- All included models had elements of care integration (such as a care coordination role, interdisciplinary collaboration and person-centred care); but the level of integration varied considerably.
- Few models of dementia-specific end-of-life care have been systematically evaluated.
- Sustainability of models of care depends on long-term funding, particularly for a dedicated care coordinator role.

**Implications for practice, theory or policy**

- Findings are relevant for developing locally responsive, integrated and person-centric models of end-of-life care for people with dementia, their families and carers.
- Recognition that dementia is a terminal disease requiring a needs-based approach to care is key to developing a model of integrated palliative care for people with advanced dementia.
- Long-term planning is needed by policy makers and health service managers to support ongoing investment in sustainability and evaluation of models of care.

## Introduction

People with dementia have different care and support needs at end of life compared to people with other life-limiting illnesses.^1,2^ People with advanced dementia may not be able to communicate their needs for pain management, assistance with eating and drinking or spiritual support.^2^ Hospitalisation is also common for people with end-stage dementia who experience symptoms including confusion, infection, pain, constipation and loss of appetite.^3^ A study of nursing home deaths across six countries found that between 60% and 83% of residents had a diagnosis of dementia, and a higher Quality of Dying in Long-Term Care score for all residents was associated with, among other factors, absence of dementia (*p* = 0.001).^4^

Quality end-of-life care is a fundamental human right for all, including people with dementia. However there is evidence that either people with dementia were not routinely referred for palliative care or that the end-of-life care models being used in aged care settings were not responsive to the needs of residents with dementia and their family or carers.^5,6^ For example, a recent retrospective study of 5804 people diagnosed with dementia in the United Kingdom, who died between 2016 and 2019, found that only one in three were identified as having palliative care needs in their last year of life.^7^ This is despite evidence of potential benefits of a palliative approach to end-of-life care for people with dementia,^8^ and the European Association for Palliative Care’s inclusion of dementia palliative care as an emerging specialty area in its recent review of standards and norms for palliative care in Europe.^9^

One reason for the low rate of referral of people with dementia to palliative care services is that dementia is often not recognised as a terminal illness.^2,5,10,11^ Another argument is that few models of end-of-life care have been developed specifically for people with advanced dementia, despite strong consensus on the optimal elements of palliative care for people with dementia.^8,12^ The impact of dying and death without quality or dignity has a profound negative effect on not only the person, but also their family and healthcare professionals.^13^

This rapid review aims to identify existing models of end-of-life care which are specific to people with advanced or end-stage dementia. The terms palliative care, and end-of-life care are challenging to separate and definitions for what constitutes end-of-life for persons with dementia are varied.^14^ In this review, palliative care includes both a palliative approach to care from all care providers (as defined by Dementia Australia^15^), delivered at any time the person with dementia has need of it during their illness, and/or a specialist palliative care service at the end of life (when death is judged as likely to occur within 12 months).^15^

A model of care is defined as a multi-dimensional concept which defines the underlying principles and core components of healthcare and other services delivered to patient or population groups, including how a person might access and travel through such services.^16,17^ An integrated model of care for people with advanced dementia was identified by the Central Coast Local Health District (CCLHD) in New South Wales, Australia, as a local priority. A research team from CCLHD was supported by a grant to develop a co-designed model of care for people with advanced dementia and their carers. A rapid review to identify existing models was chosen as it offers an efficient approach to identify and summarise existing published evidence in a timely and cost effective manner.^18^ Findings of the review, together with results of a clinical audit and a survey of bereaved carers of people who died with dementia, supported the development of a model of care.

## Methods

### Design

A rapid review methodology was used to identify published articles and grey literature to answer the question, what models of integrated, palliative care currently exist for people in the advanced and end-stages of dementia? As the main purpose of the review was to summarise information about existing models of care, quality appraisal of the evidence sources identified was not included and a narrative summary of the findings was planned.^18^

### Inclusion and exclusion criteria

The inclusion and exclusion criteria are listed in Table 1 below.

**Table 1.** Inclusion and exclusion criteria.

	Inclusion criteria	Exclusion criteria
Population	People with dementia in the last year of life, or the palliative phase of the disease, i.e. advanced, severe or end-stage dementia and/or person assessed as meeting level 6/7 of the Global Deterioration Scale.^19^	People with early or mid-stage dementia.
Setting of care delivery	All settings including acute care, community care, residential aged care, hospice and home; national and international locations.	Nil
Nature of the intervention	A care intervention, variously referred to as a model of care, programme, care pathway, care plan or framework, with multiple components demonstrating care coordination.^16,17^	Single interventions not demonstrating coordination of care.
Study design	Case studies, pilot trials, randomised controlled trials, feasibility studies, impact and process evaluations, cohort studies, economic evaluations.	Systematic, narrative, realist and other types of literature reviews.
Characteristics of sources	Articles, reports, websites or other sources in English published between 2000 and 2022.	Sources not in English, published before 2000, or not available in full text.

### Search strategy

A six-step search strategy was used: (1) an initial scoping search was run in PubMed, resulting in a test set of 20 citations to articles assessed by review team members as relevant to the topic; (2) the articles’ PubMed unique identifiers were entered into the PubMed PubReminer (https://hgserver2.amc.nl/cgi-bin/miner/miner2.cgi) open source data mining tool and a frequency table of Medical Subject Headings (MeSH) and keywords was produced; (3) the frequency table then informed development of a logic grid of MeSH terms and keywords relevant to the concepts of advanced dementia, palliative care and models of care; (4) MeSH terms and keywords were then used separately and in combination with Boolean operators (e.g. OR, AND) to search the Medline (Ovid) database; and (5) the search strategy was then adapted for the following databases: Embase (Ovid), CINAHL (EBSCO), PsycINFO (Ovid), ProQuest, Informit Collection, Joanna Briggs Institute, The Cochrane Library, The Campbell Collaboration and Google Scholar. Searches were run in April 2021 and updated in November 2021 and April 2022. The final step (6) used citation tracking, reference lists, Google Scholar, Google advanced searching and hand searching of key journals to identify further relevant sources. The search strategy for the Medline (Ovid) database is provided in Supplemental File 1.

### Study selection

Retrieved references underwent up to three stages of screening using title and abstract, with the initial screening performed by a single reviewer (SL). A smaller number of articles (*n* = 264) were then reviewed by two independent team members (two out of SL, NG, SC, SJ, DD and CC) for full-text retrieval. Any disagreements were resolved via further screening by a third reviewer (ZT) or, in a few cases, the review team. The selected articles were then divided between team members for full-text screening and selection. The review team included healthcare professionals working in palliative, end-of-life and dementia care contexts, social scientists experienced in conducting systematic and literature reviews and a senior health librarian. Additional articles identified during full text review also underwent multiple screening processes to arrive at a final set of articles for inclusion in the review.

### Data extraction and analysis

Data extraction and analysis took place in three stages. First, data from included studies were extracted by all authors in a template developed by the review team (Supplemental File 2) and then three reviewers (SL, CC and ZT) summarised in table format data including citation details, context (country, delivery setting), intervention and funding details and whether studies or models had been evaluated.

Next, the European Association for Palliative Care framework for optimal palliative care for older people with dementia^12^ was used as a best practice guide against which the included models of care were mapped. The framework has successfully been used for conducting quality appraisals and mapping palliative care domains.^8,20^ We adapted the framework in the following ways: (1) the first domain, which considers the appropriateness of palliative care for advanced dementia, was removed because models of end-of-life care for people with dementia are based on the premise that advanced dementia requires palliative care; (2) domains 2 and 3 (relating to person-centred care, shared decision-making, setting care goals with the person with dementia and their family and advance care planning) were combined into a single domain as these often overlap in practice; (3) domains 6 and 7 (relating to avoiding burdensome treatment and optimising treatment to provide comfort), were combined into one domain; we expanded domain 10 (relating to education of the healthcare team) to include other workforce issues such as communication, collaboration and barriers to integration. The adapted European Association for Palliative Care framework with nine domains is shown in Table 2.

**Table 2.** Dementia-specific end-of-life model of care framework: 9 domains^a^.

Domain (ref)	Components and care considerations
Person-centred care (D1)	Person-centred care, communication and shared decision-making regarding care goals and advance care planning
Family care, grief and support (D2)	Family care and support, communication and involvement in care planning; uncertainty, grief and bereavement
Continuity, coordination and integration of care (D3)	Continuity, coordination and integration of care
Interdisciplinary collaboration and communication (D4)	Interdisciplinary communication and collaboration, organisational and workforce issues including education of care professionals, barriers to integrated care delivery
Education (D5)	Education of the patient, family and carers
Symptom and comfort care (D6)	Optimal treatment of symptoms and providing comfort (e.g. pain, challenging behaviour); avoidance of overly aggressive, burdensome or futile treatment (e.g. nutrition/ feeding decisions); quality of life
Prognostication, death and dying (D7)	Prognostication and timely recognition of dying; preferred place of death
Psychosocial and spiritual support (D8)	Psychosocial and spiritual support
Ethical and practical issues (D9)	Social, legal, ethical, financial, practical (e.g. transport, equipment) issues

a Adapted with acknowledgment from the European Association for Palliative Care 11-domain framework for optimal palliative care for older people with dementia.^12^

Finally, the extracted data were presented in a narrative summary which highlights four main issues critical for rapid translation of findings from the review to inform development of a local model of care.

## Results

An overview of the included models of care is provided, followed by a narrative summary organised according to the following issues: referral and admission to care, integration of care, evaluation and sustainability.

### Study selection

**Figure 1.**
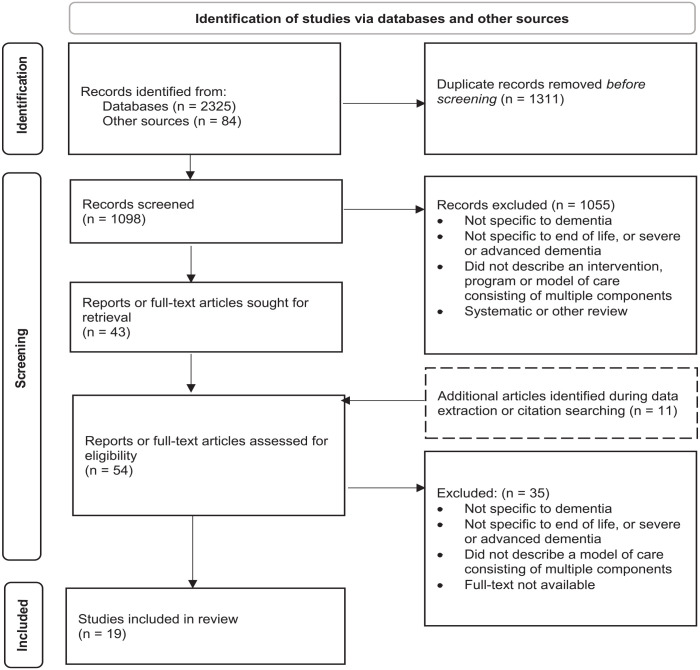
PRISMA diagram: identification, screening and selection of articles. Source: Adapted From: Page et al.^21^.

A total of 1098 records resulting from searches of databases and other sources were screened at title/abstract stage; a total of 54 full text articles and reports were assessed for eligibility; and a final set of 19 studies describing 12 models of care was selected for inclusion in the review. The PRISMA^21^ flowchart (Figure 1) summarises the study identification and selection process.

### Description of included studies

All the included models of care were complex interventions comprising multiple components and services delivered by a range of healthcare professionals. The key components of each model of care are described in Table 3. Delivery settings for the models of care included residential aged care (*n* = 5),^13,22–29^ hospice (including hospice in the home; *n* = 5),^28–33^ community and home-based care (*n* = 9),^3,13,27–29,31–37^ primary care clinics (*n* = 2),^28,29,37^ and hospital (acute care; *n* = 4)^3,13,27–29,35,36^settings. Some models were delivered in multiple settings. Four models of care were located in England,^23–26,32,33,37^ three in both Australia^22,27,34^ and the United States,^13,28–30,38^ and one each in Singapore^3,35,36,39^ and Wales.^31^

**Table 3.** Key components of models of care.

Model of care	Author/country	Setting	Referral pathway	Key components	Healthcare professionals	Funding
1. Aliviado Dementia care	Lin et al. (2022)/US	Home hospice programme delivered in suburban area	• PWD were identified from hospice medical records.	• Dementia care training for HCP • A toolbox consisting of assessment instruments, symptom management algorithms, care plans and carer education sheets • Clinical workflow changes to integrate toolbox materials into hospices’ electronic health records • Mentorship through the Aliviado technical support centre	• Nurses • Social workers • Chaplains • Ordering providers (physicians, nurse practitioners and physicians assistants) • Home health aides	Pilot funded by the National Institute on Aging at the National Institutes of Health.
2. Alzheimer’s Disease and Related Dementias Palliative Care (ADRD-PC) programme	Hanson et al.^13^/US	Hospital, home, RAC	• PWD were aged 65 years or older, identified during hospital admission with acute illness, diagnosis of dementia Stage 5–7 on the Global Deterioration Scale (GDS) and had an identified family decision-maker	The following support is provided while hospitalised and 2 weeks post discharge: • PC consultation • Phone support • Informational booklet for families • Individualised recommendations for PC domains • Assistance with completing a Medical Orders for Scope of Treatment (MOST) order set, the North Carolina version of Physician Orders for Life Sustaining Treatment (POLST) • Recommended referrals to post discharge services • Transitional care training session to PC physicians and nurse practitioners	Primary care physicians and nurse practitioners	Pilot funded by NIA R21AG052140 and National Palliative Care Research Center. Trial funded by $4.1 million dollar grant from the National Institute on Aging (NIA).
3. Challenge Pathway	Harrop et al.^31^/ Wales	Hospice, home, community	• Open referral system.	• Specialist care • Home-based hospice care • Social workers • Dementia day programmes • Peer-support groups • Training for health and social care workers, families and volunteers	• Nurse specialist • Dementia support worker	Evaluation funded by Aneurin Bevan University Health Board Research and Development Department and South East Wales Academic Health Science Partnership (SEWAHSP). Funding support was also provided via a Marie Curie Cancer Care core/programme grant, Marie Curie Research Centre, Cardiff University
4. Collaborative Model for End Stage Dementia	Lindsay^34^/Australia	Dementia day therapy unit, home	• Specialist Geriatricians	• Carer education and support • Identification of EOL • Coordination of support with GPs and PC • Post-bereavement support	• Case manager • Psychologist • Nurse • Diversional therapist	Programme funded by New South Wales Health (Illawarra Shoalhaven Local Health District)
5. Compassion Intervention	Elliott et al.^23^/England; Jones et al.^24^/EnglandJones et al.^25^/England; Moore et al.^26^/England	RAC	• Care home staff are trained to identify eligible residents (a diagnosis of moderately severe dementia in addition to another concern such as persistent distress or another condition)	• An interdisciplinary care leader who coordinates care • Education, training and support for all involved in caring for the person with dementia, particularly care home staff and carers	• Interdisciplinary care leader • Clinical lead professional responsible for medical care • RAC nurse unit manager • Other healthcare staff who deliver direct care to the person with dementia; • The wider team providing support as needed	Programme and evaluation funding was provided by Marie Curie Cancer Care (now Marie Curie) through a process administered in partnership with Cancer Research UK.
6. Model of Multidisciplinary Palliative Care (MMPC)	Abbey et al.^22^/Australia	RAC	Existing residents of RAC identified as follows: • Diagnosis of dementia • Incontinence • Functionally non-verbal or making noises indicating distress • Reduced interest in food/weight loss • Poor skin integrity • Poor peripheral circulation • Judged by experienced nursing staff as likely to die within 12 months	• Case conferencing • Education of RAC staff • Involvement and support of carers	• RAC staff • Volunteer primary care practices	Trial funded by the Prince Charles Hospital Foundation, Queensland.
7. The Nightingale Programme	Littledyke and Davis^27^/Australia	Community, residential, acute care	• Dementia Australia National Helpline • Healthcare professionals • Primary care providers • Carers/family • RAC staff • Community service providers	• Weekly case conference with a Consultant Geriatrician • Symptoms and medication management • Clinical report is produced and shared with family, GPs and other specialists and health care providers • Use of validated assessment tools • Carer education and support	• Clinical nurse consultants • Consultant geriatrician	Programme funded by the Rosemary Foundation for Memory Support
8. Oxleas Advanced Dementia Service	Sonola et al.^33^/England	Home	• Community matrons • Consultant psychiatrists • Advanced practice nurses • GPs • District nurses • Hospices • Mental health wards	• Personalised care plans • Care coordination • Carer support	• Consultant geriatric psychiatrist • Specialist nurses • Specialist social worker	Funding from clinical commissioning groups (CCGs) and local authorities/councils
9. Palliative Care in Dementia Project	Scott and Pace^32^/England	Home, RAC	• GPs • RACF nurse/manager • Psychiatrists • Alzheimer’s Society • Care manager • Hospital clinician	• Specialist palliative care worker • Educational programme for RAC staff • Healthcare workers and professionals • Referrals to other services	Specialist palliative care nurses	Programme and evaluation funded by the King’s Fund, UK
10. PEACE (University of Chicago portion)	Diwan et al.^38^/US; Shega et al.^28^/US; Shega et al.^29^/US	Primary care clinic, hospital, RAC, hospice, home	• Physicians	• Physical and psychological symptom management • Advance care planning • Education on the disease process • Community resources • Coordination of care • Patient and carer-centred care • Hospice services	• Nurses • Social workers • Fellowship-trained physicians (geriatrics) • Other disciplines as needed, eg audiology, neurology	Programme funded by The Robert Wood Johnson Foundation’s ‘Promoting Excellence in End-of-Life Care Initiative’
11. Programme Dignity	Hum et al.^3^/ Singapore; Hum et al.^35^/Singapore; Pereira et al.^36^/ Singapore	Hospital, home	• Geriatric and palliative inpatient wards • Outpatient clinics	• Regular home visits and phone calls from multidisciplinary team • Assessment and pharmacological interventions to manage pain and behavioural issues • Carer education and support through counselling and referrals to other community services • After hours support • Direct admission to ward or hospice as required	• Doctors • Nurses • Medical social workers • Hospital physicians	Research funded by the Temasek Foundation. Beyond the research project, programme is funded by the Singapore government (40%) and fundraising and donations (60%).
12. SEED Programme	Robinson et al.^37^/England	Primary care	• Patients identified from the practice dementia register and screened by a GP	• Timely planning discussions • Recognising end of life and providing supportive care • Co-ordinating care • Effective working with primary care • Managing hospitalisation • Continuing care after death • Valuing staff and ongoing learning.	Dementia nurse specialist	Programme and evaluation funding was provided by the Programme Grants for Applied Research programme (National Institute for Health Research)

EOL: end of life; GP: general practitioner; PC: palliative care; RAC: residential aged care (includes nursing homes).

The study designs used to test or evaluate the models included a randomised controlled trial,^37^ a pilot trial,^13^ feasibility studies,^23,28–30,37^ impact and process evaluations,^31,33,37^ and a prospective cohort study.^3,35,36^ Qualitative, quantitative and mixed methods were used in the included papers (e.g. surveys, interviews, patient chart/ medical record audits, field notes, ethnographic observations, case study). Some models of care were described in a single paper; other studies provided detailed descriptions and evaluations of care models in two or more papers and reports, implementation manuals and/or online websites. Two models had not been systematically or formally evaluated.^27,34^ See Table 4 for a summary of evaluation of the included models of care.

**Table 4.** Evaluation of models of care.

Model of care and study design	Primary and secondary outcomes	Methods	Results
1. Aliviado Dementia Care Feasibility, applicability and fidelity pilot	Primary: Hospice staff outcomes include dementia knowledge, confidence and attitudes Secondary (Person with Dementia): Hospital admissions and entry to nursing home, antipsychotic use, caregiver satisfaction	Pre and post training survey Informal focus group	*N* = 72 hospice staff, 11 Persons with Dementia Programme feasibility (programme completion), applicability (indication of implementing changes to practice) and fidelity (completion of assessment instrument or care plan) exceeded pre-established criteria.
2. Alzheimer’s Disease and Related Dementias Palliative Care (ADRD-PC) programme Pilot RCT completed. Multisite RCT ongoing	PO was hospital or emergency department visits in the 60 days after discharge SO patient and carer-centred were: • Patient comfort • Carer distress • PC domains addressed in the treatment plan • Access to hospice or community-based PC SO decision-making were: • Discussion of prognosis • Discussion of goals of care • Completion of MOST order set • Documented decisions against rehospitalisation or other potentially burdensome treatments	Carer interviews Chart and medical record review	*N* = 62 Persons with dementia and carer dyads • The intervention proved feasible • Hospital and emergency department visits did not differ • Intervention patients and families were more likely to: ○ Have PC domains addressed ○ Receive hospice ○ Discuss prognosis and goals of care ○ Have a MOST at 60-day follow-up ○ Make decisions to avoid rehospitalisation.
3. Challenge Pathway Implementation and impact evaluation of service outcomes for the first 16 months of operation	• Number of referrals for patients with dementia • Type of referrals • Carer knowledge, confidence and service outcomes • Healthcare professionals service impact and knowledge	Clinical data audit Interviews with project staff Surveys with carers and healthcare professionals	*N* = 3 project staff, 15 carers and 20 health care professionals • Referrals increased • There were more dementia patients referred for PC than for EOL support • Most carers and healthcare professionals rated the service as helpful
4. Collaborative Model for End Stage Dementia Case study	NR	NR	*N* = 1 Person with dementia and carer dyad Positive benefits identified
5. Compassion Intervention Multisite feasibility study	• Compare the operation of the Intervention in different health and social care settings • Estimate the cost of employing an Interdisciplinary Care Leader (ICL) • Demonstrate the Intervention caused no physical or psychological harm to person with dementia or carers	Interviews with carers and HCPs Field notes Clinical data	*N* = 9 Persons with dementia, 28 HCPs • The interdisciplinary care leader role was found to be feasible, with reasonable costs • The manual was found to be holistic and transferable to other contexts • It was not possible to assess whether the intervention led to better outcomes for residents and their families or carers, but it did no harm
6. Model of Multidisciplinary Palliative Care (MMPC) Trial in two RAC facilities	NR	Clinical data audit Documentation of programme goals and outcomes Ethnographic observation and photography Interviews with HCPs and carers Pre and post surveys with RAC staff	*N* = 17 Persons with dementia • Increased reporting of symptoms for the nine patients who died during the study • Multidisciplinary case conferencing was successful in formalising discussion of issues • No change in carer scores • Most bereaved carers expressed satisfaction with care • Staff belief in their ability to improve care increased but confidence decreased
7. The Nightingale Programme	NR	NR	NR
8. Oxleas Advanced Dementia Service Impact evaluation	• Cost of hospital admissions avoided • Patient quality of life • Carer stress levels	• Internal audit • Patients and carers questionnaires • Carer focus groups	• QUALID scores improved or remained stable over time for most • RSS scores improved or remained stable for all carers over time. • Carer reports were generally favourable
9. Palliative Care in Dementia Project Pilot study and second-stage cohort study	• ED admissions • Patient referrals to other agencies or full hospice service • Symptomatology profiling and identification of patients with dementia who need palliative care • Delivery of educational programme (carers/healthcare professionals/nurses). • Consultation/discussion held with carers	Clinical and administrative data	*N* = 50 Persons with dementia • GSF criteria are a useful prognostic tool for identifying patients with dementia who require palliative care. • Specialist palliative care nurses can, with consultant supervision, provide effective symptom control, care and support and education. • Many of end-of-life care issues reflect a lack of knowledge, expertise and understanding about disease progression (e.g. dementia) and care of people who are dying • More education is needed
10. PEACE (University of Chicago portion) Feasibility single-site pilot	• Patients and carers matched with available resources • Better utilisation of community resources • Greater use of hospice services	Interviews with patients and carers at enrolment and every 6 months following. Carers interviewed after death. Patient chart review.	• *N* = 150 patients/carer dyads • High satisfaction rates • Patients enrolled in hospice programmes were significantly more likely to die in their place of choice • Carers continued to experience significant stress
11. Programme Dignity Prospective cohort study	• Patients’ symptoms and quality of life • Caregiver burden • Other data such as comorbidities	Chart review	Patient outcomes: • A statistically significant improvement was observed in all symptom scores (pain, nutrition and neurospsychiatric symptoms) and in caregiver burden after 12 months. • A statistically significant improvement in quality of life scores was also observed. Healthcare utilisation: • One-year costs were estimated for Programme Dignity on a per patient-month basis retrospectively for the cohort enrolled in the programme (*n* = 184) and a control group (*n* = 139). • Other healthcare utilisation costs (ED visits, admissions, LOS) were also calculated. • Full enrolled Programme Dignity patients were less likely to visit ED, be admitted to hospital and had lower cumulative LOS than partially enrolled or non-enrolled patients. Cost-effectiveness: • Fully enrolled patients had lower costs at 1, 3 and 6 months than partially enrolled or non-enrolled patients (found to be statistically significant). • The cost of informal caregiving was not factored in to the economic analysis.
12. SEED Programme Multisite feasibility RCT and process evaluation	• An appropriate primary outcome measure for the intervention was not identified • Feasibility of recruitment, participant retention and 12-month follow-up for multi-centre RCT • Feasibility and acceptability, extent to which SEED intervention implemented in practice and factors influencing intervention implementation (process evaluation) • Feasibility and acceptability of available outcome measures, feasibility of capturing resource use and HR-QoL data for people with dementia and family carers, how to capture data on future care planning	Interviews, observations, dementia nurse speciality activity logs	*N* = 44 Persons with dementia • All seven components of the intervention were delivered (either at home or in care home); all were found relevant and no additional components were identified • Implementation issues included the qualifications and training needed to perform the Dementia Specialist Nurse role, lack of clarity about focus and content of the SEED intervention, the SEED intervention mostly being seen as complementary to existing services and the temporary nature of the SEED intervention affecting commitment from participating GP practices

ED: emergency department (hospital); EOL: end-of-life; ICL: interdisciplinary care leader, GSF: gold standards framework; GP: general practitioner; HCP: health care professional, HR-QoL: health related quality of life; LOS: length of stay, MOST: medical orders for scope of treatment; NR: not reported; PC: palliative care; PO: primary outcome; QUALID: quality of life in late-stage dementia (Weiner et al., 2000^58^), RAC: residential aged care (includes nursing homes); RCT: randomised controlled trial; RSS: Relative Stress Scale (Ulstein et al., 2007^59^); SO: secondary outcome.

### Domains and components of end-of-life care

Analysis and mapping of the included models of care against the nine domains of optimal palliative care for people with dementia showed that not all domains were consistently met (see Table 5). The domain for which there was the least demonstrated evidence was ethical and practical issues (D9), followed by psychosocial and spiritual support (D8) and person-centred care (D1). For some models of care, the supporting evidence for each domain was minimal or difficult to identify.

**Table 5.** Included models of care mapped to the nine domains of palliative care.

Model of care (context)	**D1**	**D2**	**D3**	**D4**	**D5**	**D6**	**D7**	**D8**	**D9**
1. Aliviado Dementia Care (New York, USA)	X	X	X	X	X	X	X		
2. Alzheimer’s Disease and Related Dementias Palliative Care (ADRD-PC) programme (North Carolina, USA)	X	X	X	X	X	X	X	X	X
3. Challenge Pathway (South Wales, UK)	X	X	X	X	X	X	X	X	X
4. Collaborative Model for End-Stage Dementia Care (NSW, Australia)	X	X	X	X	X	X	X	X	X
5. Compassion Intervention (North London, UK)	X	X	X	X	X	X	X	X	X
6. Model of Multidisciplinary Palliative Care (MMPC) for Residents with End-Stage Dementia (QLD, Australia)	X	X	X	X	X	X	X	X	X
7. Nightingale Programme (SA, Australia)	X	X	X	X	X	X	X	X	X
8. Oxleas Advanced Dementia Service (UK)	X	X	X	X	X	X	X	X	X
9. Palliative Care in Dementia (UK)		X	X	X		X	X	X	
10. Palliative Excellence in Alzheimer Care Efforts (PEACE; Chicago, USA)	X	X	X	X	X	X	X	X	X
11. Programme Dignity (Dignity in Advanced Dementia or DIADEM; Singapore)		X	X	X	X	X	X		
12. Supporting Excellence in End-of-life care in Dementia (SEED) programme (UK)	X	X	X	X	X	X	X	X	X

D1: person-centred care; D2: family care, grief and support; D3: continuity, coordination and integration of care; D4: collaboration and communication; D5: education of the person, family and carers; D6: symptom and comfort care; D7: prognostication, dying, death; D8: psychosocial and spiritual support; D9: ethical and practical issues.

Practical examples of how each of the domains of care was demonstrated in the included models were limited for the following reasons:

- lack of detail in the reporting of some models of care, for example the Challenge Pathway^31^ and the Collaborative Model for End-Stage Dementia^34^;
- resources were not freely available, for example the Aliviado Dementia Care resources^40^; or
- resources no longer exist, for example the Massive Open Online Course (MOOC) developed for carers of people with advanced dementia as part of the SEED Programme.^37^

However we have selected practical examples of how the domains of care were demonstrated, which may be useful to readers seeking to develop a locally-responsive model of care for people with advanced dementia (see Table 6).

**Table 6.** Selected practical examples of how domains of care were demonstrated in the models of care.

Domain	Examples demonstrated in models of care
(D1) Person-centred care including communication and shared decision-making regarding care goals and advance care planning	The Oxleas service^32^ has no standardised care package for patients with advanced dementia. Rather, care is tailored to each person based on their primary needs and available services. The care assessment examines the mental, physical and social needs of the person, their personal and social background and end-of-life and spiritual wishes.
(D2) Family care and support, communication and involvement in care planning; uncertainty, grief and bereavement	The Oxleas Advanced Dementia Service provides support to families and carers with the goal of enabling them to care for the person with dementia at home, pain-free and comfortable, until the end of life, with bereavement support also provided. Key to carer support is the recognition that they may be experiencing anticipatory grief due to the nature of dementia. Of nine family carers who participated in evaluation, all experienced stable or decreased scores on the Relative Stress Scale between entry into the service and 1 year, or death of the care recipient.^32^
(D3) Continuity, coordination and integration of care	The Compassion Intervention Manual^24^ provides guidance on the interdisciplinary care leader role including professional background, level of clinical experience, minimum skills and training required, factors determining case load and range of duties. The SEED Programme dementia nurse specialist role job description is provided in Appendix 3 of the SEED report,^36^ and the Northumbria Healthcare Trust job description of the same role is provided in Supplemental Material. Detailed guidance for commissioning good-quality, community-based end-of-life care in dementia is provided in the SEED report^36^; although this is UK-specific, general principles are transferable to other contexts.
(D4) Interdisciplinary collaboration and communication, organisational and workforce issues including education of care professionals, barriers to integrated care delivery	The PEACE Programme^27,28^ provides people with advanced dementia and their carers access to a range of healthcare professionals at one clinical site, including physicians (geriatrics, neurology, psychiatry, ophthalmology, dentistry), social worker, clinical nurse specialists, dietitian, audiologist, physical and occupational therapists, as well as some diagnostic tests. Both the SEED Programme^36^ and the Model of Multidisciplinary Palliative Care (MMPC)^21^ for residents with end-stage dementia, delivered in residential aged care settings, included post-death facilitated reflections (bereaved carer and care home staff – SEED Programme; care home staff only – MMPC). For care home staff, the aim of these reflections was to identify what was done well and what could have been improved in the care of the person who had died.
(D5) Education of the patient, family and carers	The Challenge Pathway^30^ provides education to family members and carers (as well as health and social care professionals and volunteers) on dementia, end-of-life care, advance care planning, emotional support and linking with local services such as the Alzheimer’s Society. Current and former carers (*n* = 15) were surveyed; all but one rated the service extremely or quite helpful; 11 indicated improvements in knowledge, confidence and practical skills. Free text responses emphasised an increase in feelings of improved safety (24-hr telephone support available) and reduced feelings of isolation.
(D6) Symptom and Comfort Care, including optimal treatment of symptoms and providing comfort (eg pain, challenging behaviour); avoidance of overly aggressive, burdensome or futile treatment (e.g. nutrition/feeding decisions); quality of life	For people with dementia enrolled in the Nightingale Programme,^26^ assessment (Functional Assessment Staging Test and Abbey Pain Assessment primarily) is repeated regularly and nursing recommendations for symptom management and co-morbidities are discussed in relation to goals of care and demonstrated for carers. Targeted and, importantly, anticipatory advice, education and support are provided to the person with dementia, their family and carers. A case study included in the description of the Oxleas Advanced Dementia Service^32^ highlights optimal treatment of symptoms for one person with advanced dementia (hydration, nutrition, safe swallowing, incontinence management, prevention of urinary tract infections, pressure area management). The person was enabled to remain at home cared for by their spouse, until they died.
(D7) Prognostication and timely recognition of dying; preferred place of death	In addition to evaluation of Programme Dignity, researchers also developed and validated a prognostic model for 6-month and 1-year mortality in home-dwelling patients with advanced dementia.^38^ The model, Palliative Support DEMentia Model (PalS-DEM), is based on 6 variables including age, dementia etiology, Functional Assessment Staging Test stage, Charlson Comorbidity Index scores, Australian National Sub-Acute and Non-Acute Patient palliative care phase and 30-day readmission frequency for the prediction of 1-year mortality. The PalS-DEM was found to be useful in identifying people with dementia at high risk of death in the next year.
(D8) Psychosocial & spiritual support	A case study of care provided by the Carunya community dementia care service (Collaborative Model for End-Stage Dementia)^33^ illustrates the importance of the spiritual needs of the person with dementia and their family and carer. In this case, the deep spiritual/emotional connection between the person with dementia, their wife (also their primary carer) and the dairy farm in rural NSW, Australia where they had lived and worked for over 50 years played a significant role in decision-making regarding end-of-life care and place of death.
(D9) Ethical, social, legal, financial, practical (e.g. transport, equipment) issues	A case study included in the description of the Oxleas Advanced Dementia Service^32^ describes the range of equipment (hospital bed, pressure mattress, sliding sheet, recliner chair with pressure cushion, hoist, incontinence pads) provided to a carer of someone with advanced dementia living at home.

### Referral and admission to care

The included models of care demonstrate a range of referral pathways and assessment for admission to a palliative care service or programme (see Table 3 for details). People with dementia were admitted to the models of care based on criteria that were often described in general terms, for example being likely to derive benefit from the intervention. In a few cases criteria were provided in more detail. For example, inclusion in the Model of Multidisciplinary Palliative Care (MMPC) programme depended on a number of the following admission criteria: existing resident of a residential aged care facility, diagnosis of dementia, incontinence, functionally non-verbal or making noises indicating distress, reduced interest in food/weight loss, poor skin integrity, poor peripheral circulation, judged by experienced nursing staff as likely to die within 12 months.^22^ Evaluation of Programme Dignity included development and validation of a prognostic model (the PalS-DEM model) for 6-month and 1-year mortality in people with advanced dementia living at home.^39^ None of the included studies reported any analysis regarding accuracy of prognostication, in other words, whether the study/programme participants were appropriately identified for the interventions delivered, and whether they were admitted into the model of care at the appropriate time based on their level of need.

### Integration of care

All included models had elements of integrated care (such as a care coordination role, interdisciplinary collaboration and person-centred care); but the level of integration varied considerably. Issues considered in relation to evaluating the degree of care integration provided by the models were: duration of care, location of care, care coordination role, interdisciplinary collaboration and communication and evidence of person-centred care.

#### Duration of care

Duration of care varied from a brief, time-limited intervention such as the ADRD-PC programme (specialist inpatient palliative care consultation, information and referrals to post-discharge services, covering the period of hospitalisation and 2 weeks after discharge),^13^ to the majority of models in which care was provided to the person with dementia from enrolment in the programme to their death. Some models such as the Collaborative Model for End Stage Dementia^34^ and the SEED Programme,^37^ also provided post-bereavement support to families and carers.

#### Location of care

Several models of care were specific to one care location only. For example, the Compassion Intervention^23–26^ and the MMPC^22^ were developed for residential aged care (RAC), with an emphasis on education and training in both dementia care and palliative care for RAC staff. Other models, such as Aliviado Dementia Care,^30^ the Challenge Pathway^31^ and the Oxleas Dementia Service,^33^ provided hospice-in-the-home care. In contrast, Programme Dignity^3,35,36^ spans a number of locations of care, all of which may be needed during the advanced stage of dementia. In addition to a range of support provided to families caring for someone with dementia at home, the programme also provides after hours support and direct admission to acute or hospice care if needed. Importantly, all members of the team have access to patient information in the national medical health records, which are also accessed by hospital and hospice staff if the patient is admitted to either facility.

#### Care coordination role

A common inclusion in the models reviewed was the use of a dedicated Clinical Nurse Consultant, Palliative Care Nurse or Social Work Coordinator (*n* = 9), whose role was to identify and enrol people with dementia (and their family and carers) into the service; conduct assessments; communicate regularly with consultant geriatricians, specialists, general practitioners, allied health and palliative care providers (e.g. through weekly case conferences) to ensure continuity of care and assist with care planning; provide education to healthcare professionals, people with dementia, their carers and families about dementia and end-of-life issues (e.g. stages of dementia disease progression, symptom management/ comfort care, what to expect when death is approaching); and provide referrals to outside support such as bereavement counselling for carers. Some models such as the Challenge Pathway^31^ and Oxleas Advanced Dementia Service^33^ also provided access to 24/7 crisis support for carers supporting a person with dementia in their home.

#### Person-centred care

Person-centred care, defined by the Australian Commission on Quality and Safety in Health Care as ‘care that is respectful of, and responsive to, the preferences, needs and values of the individual patient’,^41^ was implied in all the included models of care. However, the level of evidence varied and clear evidence of person-centred care, including advance care planning, was not identified in either the Palliative Care in Dementia^32^ model or Programme Dignity.^3,35,36^ In many of the models of care, the main mechanisms whereby the person with dementia was included in decisions relating to their care appeared to be advance care planning, (in other words, decisions made according to goals and wishes documented in the past) and interpretation of the person’s wishes by family and carers. The review found scant evidence of supported decision-making for people with advanced dementia. The ‘supportive toolbox’ of ‘skills, strategies, techniques and resources’ required by healthcare professionals to enable people with even advanced dementia to have meaningful involvement in decision-making is an area for further research.^42^

The people with dementia receiving care within each of the included models were generally described in terms of demographic detail and symptom profile. Other information, such as culturally and linguistically diverse backgrounds, Indigenous populations or identification as LGBTIQ+, was not included. Therefore, it is not known whether these models potentially benefit diverse populations of people with dementia.

### Evaluation of models of care

Primary and secondary outcomes reported in the included studies targeted patient- and carer-level outcomes (such as symptom control, evidence of advance care planning, documentation of care goals, carer distress/satisfaction/confidence and knowledge, preferred place of death achieved); care provider outcomes (such as increased knowledge and confidence in delivering care, acceptability of changes in practice); and system-level outcomes (such as improved accuracy and completeness of clinical documentation, avoidance of hospital admission, cost of employing a care coordinator, utilisation of community resources). Few models were evaluated using large, robust study designs; most were feasibility studies^23,28–30,37^

### Sustainability

Some of the models of care included in the review, such as the SEED Programme^37^ and the MMPC,^22^ had ceased operating. Reasons for this were unclear but could be assumed to be lack of funding beyond the initial pilot phase, which was dependent on research project funding. Insufficient human resourcing and/or reduced feasibility due to changes in health care policy, guidelines and delivery systems may also have been factors. For other models of care such as the Challenge Pathway^31^ and the Collaborative Model for End Stage Dementia,^34^ it was unclear whether they were still operating.

Implementation of three models of care included production of detailed documentation which is freely available. Pro-forma documents such as assessments, work instructions, flowcharts and audit tools, were developed during the trial of the MMPC and are included as appendices in the final report.^22^ The SEED Programme report^37^ provides a range of useful documents such as a job description for a dementia nurse specialist and an activity checklist for timely end-of-life care planning discussions with people with dementia and their families. Similarly, the Compassion Intervention Manual^25^ provides detailed guidance on commissioning and implementation of the intervention, although it is unclear whether it has been implemented beyond the initial pilot in two nursing homes in North London between 2014 and 2015. Resources such as these enhance the impact of models of care by facilitating transfer of the model to other contexts.

## Discussion

The review identified relatively few models of palliative care for people with advanced dementia, and these varied considerably in setting, scope, degree of integration of services and evaluation. All models demonstrated at least some evidence of at least six of the nine domains of optimal palliative care (based on the 11 EAPC domains^12^). From the narrative summary of the included models, the following four main issues emerged as key to successful translation of the research evidence into a local pilot model of care.

### Referral and admission to care

Despite a range of referral pathways to a palliative care service or programme, the included models of care provide limited insights about when, where and how to identify people with advanced dementia who are in need of end-of-life care and to initiate such care. Where the criteria for admission were provided, people with dementia have to demonstrate a significant level of deterioration from multiple and complex symptoms to be offered interventions.^25^ Yet the burden of symptoms is not the whole picture; admission to care should also encompass the psychosocial needs of people with dementia, their families and carers.^14^ Programme evaluation of models of care for people with advanced dementia should also include investigation of referral pathways and admission criteria to identify whether these were needs-based and applied consistently. This review also showed that accurate prognostication for people with advanced dementia was not a major element of most of the included models of care. Prognostication tools to accurately identify people in need of end-of-life care (e.g. the Supportive Palliative care Indicators Tool (SPICTTM)^43^,^44^ have been developed and implemented successfully. However tools that rely on a chronological disease trajectory are less useful for people with advanced dementia, and recent research has identified lack of consistency in how end of life in dementia is defined, and called for a move beyond prognostication to a needs-based approach.^14^

### Integration of care

Traditional models of palliative care are designed to be delivered in the last 6 months of life. Given the variable disease trajectory of dementia, a longer-term palliative approach to care is needed for people with advanced dementia. A relatively brief, time-limited model of care such as the ADRD-PC programme^13^ may provide only short-term benefits for people with advanced dementia; this is acknowledged by the authors of the study. Models built around one care delivery location, for example residential aged care,^22,24,26^ run the risk of fragmenting care when transitions between care locations are required, for example an acute medical episode requiring hospitalisation.

The models that provided care from the time the person and their family/carer enrolled in the service until their death, and in some cases post-bereavement support, and included a care coordinator role, and were not specific to one care location, were better aligned with the optimal domains of palliative care for people with advanced dementia. For example, the Nightingale Programme based in South Australia,^27^ is an integrated model of care (referrals accepted from a range of sources; nursing assessment, care planning, interdisciplinary collaboration, coordination of services provided by a care coordinator; and support for carers), in which the person with dementia remains until their death, regardless of whether their care setting changes.

Best practice models of palliative care should be built around the person with dementia, regardless of their care setting, and how long they need to receive care. Most importantly, while best practice models of care should incorporate all nine domains of palliative care, they should also be flexible enough to take into account the needs of each individual, particularly regarding psychosocial and spiritual support, and needs relating to self-determination, familiarity and safety.^2,45^

This applies particularly to people with dementia who are Indigenous, or identify as LGBTIQ+, or live in rural or remote locations, or are culturally or linguistically diverse or live with a disability. While consideration of these factors should be implicit in person-centred care, it is worth noting explicitly that they may impact access to, and suitability of, dementia palliative care. For example, a recent scoping review identified a gap in the literature on dementia palliative care in rural areas, and a need for more research on whether technology can be used to mitigate barriers to accessing care.^46^

The Australian Commission on Quality and Safety in Health Care identifies one of the key dimensions of person-centred care as involvement of carers and family.^41^ Therefore the emotional, psychological, spiritual and practical support needs of family carers should be considered in any model of dementia palliative care. Ideally, families and carers should be meaningfully engaged in shared decision-making processes with healthcare professionals involved in providing care.^6^

The quality of end-of-life care and death experience is shaped by the care needs, preferences and experiences of both the person with dementia, and their carer and family.^47^ The needs of family carers and the person with dementia are often interdependent (but not always congruent).^5,48^ For example, interviews with residential aged care staff and review of case notes for people enrolled in the MMPC model of care revealed several instances in which the person with dementia had an advance care directive but their family member was reluctant for it to be followed.^22^ It is important therefore, that families and carers are appropriately engaged in care planning and considered when developing end-of-life models of care.

It could be argued that palliative care should be offered at the point of diagnosis for any life-limiting illness including dementia.

However some people with dementia may not welcome a palliative approach to their care soon after diagnosis. They may not be ready to discuss advance care plans or end-of-life decisions, or even seemingly minor issues such as eating and drinking problems.^49,50^ Models of care that continuously and gradually support people with dementia through all stages of their experience of the disease, are likely to provide the best opportunity for them to have meaningful engagement in care decisions and experience person-centred care.

### Evaluation of models of care

The wide range of outcome measures used in evaluating some of the included models of care indicates the difficulties in assessing the effectiveness of complex interventions to improve end-of-life care for people with dementia. Further research is needed to develop methodologies for evaluating the effectiveness of complex models of care in real-world settings, including (1) outcome measures for patients, carers and healthcare professionals that are targeted, easy to deliver and not burdensome^29^; (2) patient and carer experience measures that capture a more nuanced picture beyond the commonly used experience indicators of patient comfort and carer distress; and (3) quality indicators that focus on processes, outcomes and structure of care.

Evaluation of models of care should also take into account the costs of caring. Only two of the included models reported any economic evaluation measures, namely the Compassion Intervention which calculated the cost of employing a care coordinator^26^ and the Oxleas Advanced Dementia Service which estimated savings due to hospital admissions avoided.^33^ None of the models evaluated the substantial unpaid care provided by families and carers.

### Sustainability

This review found very limited evidence to identify the conditions needed to support long-term sustainability of any model of end-of-life care for people with dementia, but it can be inferred that adequate, guaranteed ongoing funding of the programme and the position of care coordinator as a dedicated role, not an addition to other, existing duties, are key. In a resource-constrained environment, some organisations may have to be selective in which components of a model of care could be implemented depending on local context and availability of existing resources. However there is clear evidence to support what a best practice end-of-life model of care for people with dementia should look like,^9,12,20^ and ideally all domains of care should be addressed in any model. At the same time, this review demonstrates that models must be context responsive to be sustainable. Models such as the Compassion Intervention^25^ and the MMPC^22^ designed for residential aged care facilities will necessarily focus on different domains than models such as the Challenge Pathway^31^ and the Nightingale Programme^27^ designed to deliver care primarily in the community. Flexibility and agility to scale up or down as a pragmatic approach to implementing models of care may be needed to improve sustainability while managing deviation from the original design of the model.^51^

## What this study adds?

This rapid review provides an overview of end-of-life models of care that are specific to the needs of people with advanced or end-stage dementia. Examples of models of care are provided, including some with useful, freely available supplementary resources. Table 3 (Key components of models of care) and Table 4 (Evaluation of models of care) may be valuable sources of information for clinicians and health service managers seeking to develop a context-specific, model of end-of-life care for people with dementia. The selected practical examples of how the domains of care were demonstrated in the included models of care may also be useful (see Table 6).

## Strengths and limitations of the study

The focus in rapid reviews is on rapidly producing high-quality evidence to support health system decision-making and policy in a time-limited and cost-effective manner^18^; hence, an inherent limitation is that some articles may not be identified during searching. Furthermore, the pragmatic decision to use one reviewer for initial screening of references may have resulted in some relevant studies being excluded at this stage.

Publication bias may also be a limitation of this review. Some relevant models of care may not have been reported and others, implemented as research projects, may have continued after the life of the project but not reported further.

Screening for relevance was challenging due to definitional issues around the concepts of end of life for people with dementia^14^ and models of care.^16^ This ambiguity was reflected in the multiple and ongoing team discussions held in reaching consensus about several models found.

Further, several models of care were identified that were still under development and therefore not included in this review. These were the Model for Palliative Care Project (Ireland; https://pallcare4dementia.com/the-project/),^52–54^ the Partnership Model of Hospice Enabled Dementia Care (Ireland),^55^ and the Empowering Better End of Life Dementia Care (EMBED-Care Programme; United Kingdom; https://www.ucl.ac.uk/psychiatry/research/marie-curie-palliative-care-research-department/research/centre-dementia-palliative-care).^56^ An evaluation of the Partnership Model was published too late for inclusion in this review.^57^ These models are noted here for monitoring and future review.

## Conclusion

This review adds to the growing body of literature on end-of-life care for people with dementia by identifying and examining models of care implemented in a range of real-world settings. We sought examples of how the substantial body of evidence on the optimal components of a model of care for this vulnerable population group had been translated into practice and, ideally, evaluated and embedded in health and social care delivery. While a comprehensive, fully integrated, appropriately funded and sustainable model of care was not found, many of the included models offer valuable evidence to assist clinicians and healthcare managers to develop and implement best-practice, context-responsive models of end-of-life care for people with dementia.

## Supplemental Material

sj-pdf-1-pmj-10.1177_02692163231171181 – Supplemental material for Models of care for people with dementia approaching end of life: A rapid review Click here for additional data file. Supplemental material, sj-pdf-1-pmj-10.1177_02692163231171181 for Models of care for people with dementia approaching end of life: A rapid review by Suzanne Lewis, Zoi Triandafilidis, Cassie Curryer, Sarah Yeun-Sim Jeong, Nicholas Goodwin, Sally Carr and Daneill Davis in Palliative Medicine

sj-pdf-2-pmj-10.1177_02692163231171181 – Supplemental material for Models of care for people with dementia approaching end of life: A rapid review Click here for additional data file. Supplemental material, sj-pdf-2-pmj-10.1177_02692163231171181 for Models of care for people with dementia approaching end of life: A rapid review by Suzanne Lewis, Zoi Triandafilidis, Cassie Curryer, Sarah Yeun-Sim Jeong, Nicholas Goodwin, Sally Carr and Daneill Davis in Palliative Medicine
