# Supplementary material for: Models of care for people with dementia approaching end of life: A rapid review
Source: Palliat Med. 2023 May 7;37(7):915–30. doi: 10.1177/02692163231171181 (PMC10336605; doi:10.1177/02692163231171181)
Supplement: sj-pdf-1-pmj-10.1177_02692163231171181 – Supplemental material for Models of care for people with dementia approaching end of life: A rapid review [file sj-pdf-1-pmj-10.1177_02692163231171181.pdf]

Supplementary file 1: Search strategy – Medline (Ovid)

1 exp Dementia/

2 "dement*".ab,ti.

3 "alzheimer*".ab,ti.

4 1 or 2 or 3

5 Palliative Care/

6 Terminal Care/

7 Hospice Care/

8 exp Advance Care Planning/

9 Attitude to Death/

10 exp Bereavement/

11 Hospices/

12 exp Life Support Care/

13 Terminally Ill/

14 Death/

15 "palliat*".ab,ti.

16 "hospice*".ab,ti.

17 "end of life".ab,ti.

18 "terminal care".ab,ti.

19 5 or 6 or 7 or 8 or 9 or 10 or 11 or 12 or 13 or 14 or 15 or 16 or 17 or 18

20 "Delivery of Health Care"/og [Organization & Administration]

21 "Delivery of Health Care, Integrated"/

22 exp Patient Care Team/

23 Patient-Centered care/ or exp Patient Navigation/

24 "model* of care ".ab,ti.

25 "care path* ".ab,ti.

26 framework*.ab,ti.

27 program*.ab,ti.

28 continuity.ab,ti.

29 seamless*.ab,ti.

30 20 or 21 or 22 or 23 or 24 or 25 or 26 or 27 or 28 or 29

31 4 and 19 and 30

32 limit 31 to (english language and yr="2000 -Current")
