# Supplementary material for: Models of care for people with dementia approaching end of life: A rapid review
Source: Palliat Med. 2023 May 7;37(7):915–30. doi: 10.1177/02692163231171181 (PMC10336605; doi:10.1177/02692163231171181)
Supplement: sj-pdf-2-pmj-10.1177_02692163231171181 – Supplemental material for Models of care for people with dementia approaching end of life: A rapid review [file sj-pdf-2-pmj-10.1177_02692163231171181.pdf]

Supplementary File 2: resource review template for included models of care

**Reference:**	
Is a model of care described?	
Is a model of care evaluated?	
To be included in the review? Y/N (include reason/s for inclusion or exclusion)	
**Context**	
• Country, region, locality	
• Target/priority population(s) - incl. CALD, indigenous	
• Care setting , eg all; community; home; primary care; acute care; hospice, etc.	
**Intervention/strategy type**	
Is an intervention or strategy described? If so, provide a brief description.	
**Model of care**	
**1.** Person-centred care? Patients, carers or families involved?	
2. Is continuity of care/ care coordination demonstrated? What are the referral pathways / access opportunities?	

3. Is care multidisciplinary and/or across sectors within health? If so, which disciplines deliver care in which settings? (eg. Specialist palliative nurses in hospices and in the home)	
4. Is there evidence of cross-sectoral collaboration, eg health, social care, not for profit groups, community groups?	
5. Is funding described? If so, how is the MoC or program funded?	
6. Are governance and accountability described? If so, what is the governance structure, how do accountability and reporting work?	
**Findings**	
Brief description	
**Evaluation**	
Has the MoC been evaluated? If so, how? Eg. Anecdotal feedback, pre- and post-program (validated scales, interviews, mixed methods), control group, matched cases and controls, cohort study, RCT, systematic review, qualitative (interviews, focus groups, etc)	
• Costs of care	

• Care experiences	
• Care outcomes	
• Utilisation of services	
• Other	
**Related articles/reports**	
If you become aware of any related articles or reports, record them here.	
Reference lists checked?	
